# Estimating the Burden of Chagas Disease in the United States

**DOI:** 10.1371/journal.pntd.0005033

**Published:** 2016-11-07

**Authors:** Jennifer Manne-Goehler, Chukwuemeka A. Umeh, Susan P. Montgomery, Veronika J. Wirtz

**Affiliations:** 1 Department of Medicine, Beth Israel Deaconess Medical Center, Harvard Medical School, Boston, MA, United States of America; 2 Department of Global Health, Boston University School of Public Health, Boston, MA, United States of America; 3 United States Centers for Disease Control and Prevention, Atlanta, GA, United States of America; Universidad Autónoma de Yucatán, MEXICO

## Abstract

**Background:**

In recent years, there has been growing awareness of the significant burden of Chagas disease in the United States (US). However, epidemiological data on both prevalence and access to care for this disease are limited. The objective of this study is to provide an updated national estimate of Chagas disease prevalence, the first state-level estimates of cases of *T*. *cruzi* infection in the US and to analyze these estimates in the context of data on confirmed cases of infection in the US blood supply.

**Methods:**

In this study, we calculated estimates of the state and national prevalence of Chagas disease. The number of residents originally from Chagas disease endemic countries were computed using data on Foreign-Born Hispanic populations from the American Community Survey, along with recent prevalence estimates for Chagas disease in Latin America from the World Health Organization that were published in 2006 and updated in 2015. We then describe the distribution of estimated cases in each state in relation to the number of infections identified in the donated blood supply per data from the AABB (formerly American Association of Blood Banks).

**Findings:**

The results of this analysis offer an updated national estimate of 238,091 cases of *T*. *cruzi* infection in the United States as of 2012, using the same method as was used by Bern and Montgomery to estimate cases in 2005. This estimate indicates that there are 62,070 cases less than the most recent prior estimate, though it does not include undocumented immigrants who may account for as many as 109,000 additional cases. The state level results show that four states (California, Texas, Florida and New York) have over 10,000 cases and an additional seven states have over 5,000 cases. Moreover, since 2007, the AABB has reported 1,908 confirmed cases of *T*. *cruzi* infection identified through screening of blood donations.

**Conclusions:**

This study demonstrates a substantial burden of Chagas disease in the US, with state variation that reflects the distribution of at risk Latin American immigrant populations. The study lends important new insight into the distribution of this disease in the US and highlights the need for further research quantifying prevalence and incidence to guide interventions for control of Chagas disease across the US.

## Author Summary

Chagas disease is a parasitic infection that primarily affects poor populations in Latin America. However, awareness of this disease in the United States has increased in recent years. In this study, we utilize data from the American Community Survey and the World Health Organization to estimate the number of cases of Chagas disease in the United States. We find that there are an estimated 238,000 cases across the United States, along with four states that each has over 10,000 cases (California, Texas, New York and Florida). We also analyze data from the United States blood donation which shows that about 1,900 cases have been identified through blood donation. We conclude that there is still a substantial burden of Chagas disease in the US, though the burden is focused in certain geographic regions. We also highlight the need for further research to better quantify prevalence and incidence in order to guide interventions to diagnose and treat patients with Chagas disease across the US.

## Introduction

Chagas disease, caused by infection with the parasite *Trypanosoma cruzi*, affects an estimated 8–10 million people globally. Among those infected with the parasite, approximately 30% will develop chronic Chagas cardiac disease, including serious arrhythmias and heart failure. This infection can also be transmitted congenitally, from infected mother to child. Though Chagas disease has historically been considered a condition of the rural poor in Latin America, in recent years there has been growing recognition of the burden of Chagas disease in the United States (US) and Europe due to human migration from Latin America.[1–3] Moreover, both autochthonous transmission in the southern US and congenital transmission have been documented, although the numbers of cases are small and the risk of transmission is undefined.[4, 5] In the United States, there is no national level surveillance for Chagas disease; the most recent study to estimate the prevalence of this disease in the US, published in 2009, concluded that approximately 300,167 individuals with Chagas disease were living in the United States in 2005.[1]

Since these estimates were published, a more recent American Community Survey (ACS) was released, allowing for an updated estimation of the numbers of *T*. *cruzi* infections at both the state and national level. In addition, screening of the blood supply was initiated in 2007 and surveillance data on cases identified in the donated blood supply since that time offer additional insight into the geographic distribution of Chagas disease in the US. The objective of this study is to provide an updated national estimate and the first state-level estimates of cases of *T*. *cruzi* infection in the US as of 2012–2013 and to analyze these estimates in the context of data on confirmed cases of infection in the US blood supply during the period from 2007–2013.

## Methods

In this study, we calculated estimates of the state and national prevalence of Chagas disease. We did not estimate possible US-acquired infections, either congenital or vector-borne. State-level estimates of the number of residents originally from Chagas disease endemic countries were computed using data on Foreign-Born Hispanic populations by state from the ACS; state totals were summed to provide a national estimate. This was compared to the national level estimate generated using data on Foreign-Born Hispanic populations for the entire nation from the ACS. To estimate the number of cases of Chagas disease we used recent prevalence estimates for the Latin American countries of origin from the World Health Organization (WHO).[6–8] The WHO has provided country-based estimates of prevalence of *T*. *cruzi* infection in 2006 and updated these estimates in 2015 to reflect the impact of national vector control programs in the intervening period. State-Level Foreign-Born Hispanic Population Estimates by single Country of Origin group were computed using the 2012 ACS data for a five-year period (2008–2012).

The prevalence by state was estimated using Hispanic Country of Origin groups for each state as estimated by the ACS. For each single Country of Origin group, an expected number of cases were computed by multiplying the estimated state population by the proportion who had migrated prior to 2010 by the respective prevalence in 2006 in the Country of Origin and adding to that the estimated population that had migrated after 2010 multiplied by the respective prevalence reported by WHO in 2015. This method is represented in the following equation:

*Estimated Cases of Chagas disease in State B from Country A Origin Group* = (*Population that migrated from Country A to State B prior to 2010* X *National prevalence of T*. *cruzi infection in Country B per WHO 2006*) + (*Population that migrated from Country A to State B after 2010* X *National prevalence of T*. *cruzi infection in Country B per WHO 2015)*

The estimated number of cases for each state was calculated as the sum of the expected number of cases for each of the single Hispanic Country of Origin groups in that state. The state totals were then added to provide a national prevalence estimate. For comparability, we also calculated the estimated number of cases at the national level in 2012 by taking the sum of the estimated number of infections for each single Country of Origin group at the national level. This method is the same as that which was used in Bern and Montgomery (see Table 1).[1]

**Table 1:** Estimated *T*. *cruzi* cases in the United States in 2012 and confirmed cases of *T*. *cruzi* infection in donated blood per AABB from 2007–2013, by state.

State	Est. Cases	AABB Cases	State	Est. Cases	AABB Cases
Alabama	1,116	8	Montana	46	1
Alaska	110	----	Nebraska	855	3
Arizona	6,440	28	Nevada	3,712	25
Arkansas	1,161	25	New Hampshire	159	3
California	70,860	707	New Jersey	8,686	32
Colorado	3,219	4	New Mexico	1,752	4
Connecticut	1,924	8	New York	17,403	160
Delaware	339	----	North Carolina	5,408	41
D.C.	745	2	North Dakota	23	1
Florida	18,096	260	Ohio	1,142	9
Georgia	5,681	37	Oklahoma	1,407	17
Hawaii	139	----	Oregon	1,995	13
Idaho	611	----	Pennsylvania	1,804	7
Illinois	9,316	22	Rhode Island	641	1
Indiana	1,705	12	South Carolina	1,486	15
Iowa	716	5	South Dakota	82	----
Kansas	1,273	9	Tennessee	1,900	14
Kentucky	618	9	Texas	36,977	176
Louisiana	1,427	15	Utah	1,767	24
Maine	49	1	Vermont	36	----
Maryland	5,926	29	Virginia	7,346	103
Massachusetts	3,346	9	Washington	3,144	18
Michigan	1,258	7	West Virginia	88	1
Minnesota	1,443	2	Wisconsin	1,239	3
Mississippi	434	11	Wyoming	112	----
Missouri	927	17	TOTAL	238,091	1,908

The AABB data included 6 confirmed infections whose state was unknown and 4 confirmed infections in Puerto Rico, not included in Table 1.

Finally, we describe the distribution of estimated cases in each state in relation to the number of infections identified in the donated blood supply per de-identified data from the AABB (formerly American Association of Blood Banks). AABB aggregates voluntary reports of donor Chagas disease testing from most but not all U.S. blood collection agencies. These data include all confirmed cases reported to them from January 2007 to September 2013. These data do not include cases diagnosed via mechanisms other than blood donation, such as community-based screening efforts or other clinical settings. [9] This study was approved by the Boston University Medical Center Institutional Review Board (IRB) (Protocol H-32356).

## Results

**Fig 1:**
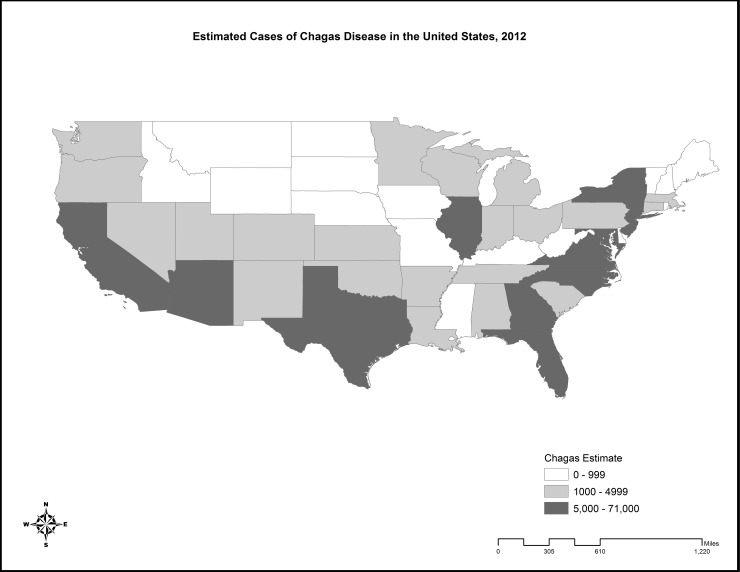
Map of estimated cases of Chagas disease in the United States, 2012.

The results of this analysis offer an updated national estimate of 238,091 cases of *T*. *cruzi* infection in the United States as of 2012 using the same method as was used by Bern and Montgomery to estimate the number of cases in 2005 (Table 1). [1] This represents a decrease of 62,070 cases from this most recent prior estimate. By comparison, summing the number of cases estimated by state offers a slightly lower national estimate of 238,072 cases. The state level results show four states with over 10,000 cases and an additional seven states with over 5,000 cases (Fig 1). Moreover, since 2007, the AABB has reported 1,908 confirmed cases of *T*. *cruzi* infection identified through screening of blood donations.

## Discussion

This study offers the first state-level analysis of the burden of Chagas disease in the US and provides objective data to inform future policies on screening and clinical care provision for this disease. This updated national estimate of 238,091 cases of *T*. *cruzi* infection represents an approximately 20% decrease in the number of infections estimated for the US in 2005, most likely due to a decrease in the estimated population of foreign-born Latin Americans based on US Census population surveys conducted after 2005. The state level estimates show a geographically focal burden with highest estimated numbers of cases in California, Texas, Florida and New York. Data on cases identified through blood donor screening is largely but not completely congruent with the state level estimates. This difference may reflect variation in community blood donation preferences related to country of origin, since blood donations are voluntary, or possibly other cultural or social differences. [10]

There are several limitations to this study. First, we focused our estimation of the cases of Chagas disease to documented foreign born immigrants from endemic countries in Latin America only. Estimates of undocumented immigrants in the United States are no longer available from U.S. Department of Homeland Security, the source of population totals by country of origin used in the previously published estimate. Recent data from the Pew Research Center suggest that in 2012 there were approximately 11.2 million undocumented immigrants in the United States. Moreover, among the leading countries of origin for the undocumented population were several countries with substantial prevalence of Chagas disease, Mexico, Guatemala and El Salvador.[11] [1]. Based on these 2012 Pew Research Center estimates of undocumented Latin American immigrants in the United States and WHO country Chagas disease prevalence, an additional 88,000–109,000 people with Chagas disease may be living in the United States, bringing the estimated total number of cases to between 326,000 and 347,000. Because of gaps in data available, we cannot provide state level estimates of Chagas disease among undocumented Latin American immigrants. A second limitation of this study was the inability to obtain reliable data to estimate both autochthonous of *T*. *cruzi* transmission and congenital transmission of *T*. *cruzi* infection among populations of Latin American origin. A third limitation of this study was that we assumed the prevalence of Chagas disease in the foreign born US immigrant population was equal to that of the country of origin. Due to a lack of data, we were unable to account for age, sex or year of immigration. Finally, for states whose population in any of the Hispanic origin groups was too small to be estimated, they were assumed to have zero members in that group and consequently no expected infections. If anything, this would result in an underestimation of the true number of cases.

These findings have important implications for health policy regarding Chagas disease in the US. The estimate from this study is similar to the previous report and suggests a continuing burden of Chagas disease in the US. This research is a critical first step in addressing Chagas disease in the United States. Other important research gaps in our understanding of the epidemiology of this disease in the US include: (1) representative studies to generate an evidence-based prevalence of disease nationwide; (2) studies to estimate the risk for acquiring *T*. *cruzi* infection via congenital or autochthonous transmission in the US; and (3) a national level definition of the contribution of *T*. *cruzi* infections to cardiac disease. Defining the Chagas disease burden in the US is necessary to inform efforts to ensure access to appropriate care and treatment among the populations at risk.

In conclusion, this study demonstrates a sustained substantial burden of Chagas disease in the US and offers the first state level prevalence estimates for Chagas disease. We also show state variation in burden, reflecting the distribution of at risk Latin American immigrant populations. The study lends important new insight into the distribution of this disease in the US and highlights the need for further research quantifying prevalence and incidence to guide interventions for control of Chagas disease across the US.

## References

[1] Bern C, Montgomery SP. An estimate of the burden of Chagas disease in the United States. Clinical infectious diseases: an official publication of the Infectious Diseases Society of America 2009; 49(5): e52–4. 19640226 10.1086/605091

[2] Bern C, Montgomery SP, Herwaldt BL, et al Evaluation and treatment of chagas disease in the United States: a systematic review. JAMA: the journal of the American Medical Association 2007; 298(18): 2171–81. 10.1001/jama.298.18.2171 18000201

[3] Manne-Goehler J, Reich MR, Wirtz VJ. Access to care for Chagas disease in the United States: a health systems analysis. The American journal of tropical medicine and hygiene 2015; 93(1): 108–13. 10.4269/ajtmh.14-0826 25986581 PMC4497880

[4] Garcia MN, Woc-Colburn L, Rossmann SN, et al Trypanosoma cruzi screening in Texas blood donors, 2008–2012. Epidemiology and infection 2014: 1–4. 10.1017/S0950268814002234 25170765

[5] Centers for Disease Control and Prevention. Congenital transmission of Chagas disease—Virginia, 2010. MMWR Morbidity and mortality weekly report 2012; 61(26): 477–9. 22763884

[6] 2008–2012 American Community Survey 5-Year Dataset. In: Bureau USC, 2014.

[7] Pan American Health Organization. Estimacion Cuantitativa de la Enfermedad de Chagas en las Americas. In: Department of Control of Neglected Tropical Diseases, 2006.

[8] World Health Organization. Chagas disease in Latin America: an epidemiological update based on 2010 estimates. World Health Organization Weekly Epidemiological Record 2015; 90(6): 33–44. 25671846

[9] AABB. AABB Confirmed Chagas Cases, Janaury 2007—September 2013. In: AABB, 2013.

[10] James AB, Josephson CD, Castillejo MI, Schreiber GB, Roback JD. Epidemiological Profiles of Foreign-Born and US-Born Hispanic Blood Donors in a Major Metropolitan Area in the United States. Journal of blood transfusion 2012; 2012: 820514 10.1155/2012/820514 24089652 PMC3771132

[11] Pew Research Center. Unauthorized Immigrant Totals Rise in 7 States, Fall in 14: Decline in Those from Mexico Fuels Most State Decreases: Pew Research Center, 2014 11 18.

